# A dataset on the phylogeny of *Mycobacterium avium* subsp. *paratuberculosis* isolated from patients in a referral gastrointestinal diseases centre in the Sudan

**DOI:** 10.1016/j.dib.2025.112257

**Published:** 2025-11-11

**Authors:** Sanaa M. Idris, Wisal A. Elmagzoub, Maha Isameldin, Nassir Arabi, Abdelmonem Abdo, Mustafa Ibrahim, Sahar M. Bakhiet, Julius B. Okuni, Lonzy Ojok, Ahmed A. Gameel, Uwe Truyen, Ahmad Amanzada, Ahmed Abd El Wahed, Kamal H. Eltom, ElSagad Eltayeb

**Affiliations:** a Institute of Animal Hygiene and Veterinary Public Health, Faculty of Veterinary Medicine, Leipzig University, Leipzig, Germany; b Department of Animal Health and Safety of Animal Products, Institute for Studies and Promotion of Animal Exports, University of Khartoum, Shambat, Khartoum North, Sudan; c Department of Pathology, Faculty of Veterinary Medicine, University of Khartoum, Shambat, Khartoum North, Sudan; d Department of Biology and Biotechnology, College of Applied and Industrial Sciences, University of Bahri, Khartoum North, Sudan; e Ibn Sina Specialised Hospital, Khartoum, Sudan; f Faculty of Medicine and Health Sciences, Omdurman Islamic University, Omdurman, Sudan; g National Centre for Gastroenterology and Liver Diseases, Ministry of Health, Khartoum, Sudan; h Department of Molecular Biology, Institute of Endemic diseases, University of Khartoum, Sudan; i College of Veterinary Medicine, Animal Resources and Biosecurity (COVAB), Makerere University, Kampala, Uganda; j Department of Pathology, Faculty of Medicine, Gulu University, Gulu, Uganda; k Department of Gastroenterology and Gastrointestinal Oncology, University Medical Centre Göttingen, Göttingen, Germany; l Faculty of Medicine, Al Neelain University, Khartoum, Sudan

**Keywords:** Sudan, *Mycobacterium avium* subsp. *paratuberculosis*, Paratuberculosis, Gastrointestinal complaints, Human isolates

## Abstract

*Mycobacterium avium* subsp. *paratuberculosis* (MAP) is the causative agent of paratuberculosis in animals, a disease characterized by chronic intestinal inflammation. MAP has also been detected in patients with Crohn’s disease and other illnesses, raising concerns about its potential public health implications. Recently, we isolated MAP from tissue and stool samples of human patients with gastrointestinal complaints for the first time in the Sudan. This dataset aimed at investigating the phylogenetic relationships of these Sudanese MAP human isolates to local animal MAP isolates and MAP strains worldwide. DNA was extracted from MAP cultures of patient tissues and stools, partially amplified for the MAP IS1311 gene using nested PCR, and then sequenced. High-quality sequences of 13 isolates (2 stool and 11 tissue isolates) together with corresponding sequences of Sudanese MAP animal isolates and of MAP strains from other countries were used to construct a phylogenetic tree. The phylogeny tree showed that the Sudanese human MAP isolates are closely related to the Sudanese animal isolates and to the MAP S-type (Sheep-type) strains from other countries including Australia, the USA, Germany, and New Zealand.

Specifications Table

**Table:** 

Subject	Biology
Specific subject area	Molecular epidemiology
Type of data	Phylogenetic tree, Tables
Data collection	DNA extraction from cultured MAP isolates, nested PCR targeting IS1311 gene, Sanger sequencing, sequence alignment and phylogenetic tree construction using Geneious programme (Geneious version 10.0, Biomatters, available from http://www.geneious.com)
Data source location	Sudan (Human and Animals Isolates)
Data accessibility	repository (https://data.mendeley.com/datasets/nwx7xgfnxp/1)
Related research article	Elmagzoub, W.A., Idris, S.M., Isameldin, M., Arabi, N., Abdo, A., Ibrahim, M., Khan, M.A.A., Tanneberger, F., Bakhiet, S.M., Okuni, J.B., Ojok, L., Gameel, A.A., Abd El Wahed, A., Bekaert, M., Mukhtar, M.E., Amanzada, A., Eltom, K.H., Eltayeb, E., 2022. Mycobacterium avium subsp. paratuberculosis and microbiome profile of patients in a referral gastrointestinal diseases centre in the Sudan. PLoS One 17, e0266533.

## 1 Value of the Data

• Isolation MAP from human patients with gastrointestinal complaints support the assumption that consider it a zoonotic pathogen.
• As the dataset provides evidence of relatedness between human and animal MAP isolates in the Sudan, it will be of interest for One Health specialists.
• These data are valuable for researchers, microbiologists, and epidemiologists in tracing transmission routes, assess cross-species infection risks.
• It is also valuable to public health workers to develop prevention and control strategies.
• Close phylogenetic relatedness of the Sudanese MAP isolates to S-type (Sheep-type) strains from Australia, the USA, Germany, and New Zealand provides a valuable information to PTB researchers, who study the origin and global distribution of MAP strains.

## 2 Background

*Mycobacterium avium* subsp. *paratuberculosis* (MAP) is a significant animal pathogen with potential public health implications. It is the causative agent of paratuberculosis (PTB), a notifiable chronic disease in ruminants [1]. Although its zoonotic potential is still under scientific debate, several studies have reported detection and isolation of MAP from both symptomatic and asymptomatic human subjects [[2], [3], [4], [5]].

Based on host association and genetic characteristics, MAP is classified into three major types: Type I (Sheep or S-type, which mainly infects sheep), Type II (Cattle or C-type, which mainly infects cattle), and Type III, an intermediate form that affects sheep, goats, cattle, and camels [6,7]. Although these types prefer specific species, cross-species transmission occurs [8] phylogenetic analyses have indicated that human-derived isolates tend to cluster closely with C-type strains [9,10].

In the Sudan, MAP infection has been documented in both large and small ruminants [[11], [12], [13], [14], [15], [16]]. Given that a significant portion of the Sudanese population is engaged in agropastoral livelihoods, involving frequent contact with livestock under limited biosecurity measures, the risk of zoonotic transmission is elevated [15,16], which can be supported by our report of the detection of MAP DNA and isolation of viable MAP from tissue and stool samples of Sudanese human patients with chronic gastrointestinal complaints [17], marking the first confirmed human infection in the country. The present data builds upon our previous report by conducting a phylogenetic relationship of these human MAP isolates to Sudanese animals isolates as well as to MAP strains worldwide.

## 3 Data Description

This dataset comprises partial high-quality sequences of MAP IS1311 gene of 13 Sudanese human patient isolates (11 from tissue and 2 from stool samples), which were used to determine their phylogenetic relationship to MAP isolates obtained from Sudanese animals and to MAP strains worldwide.

**Fig. 1:**
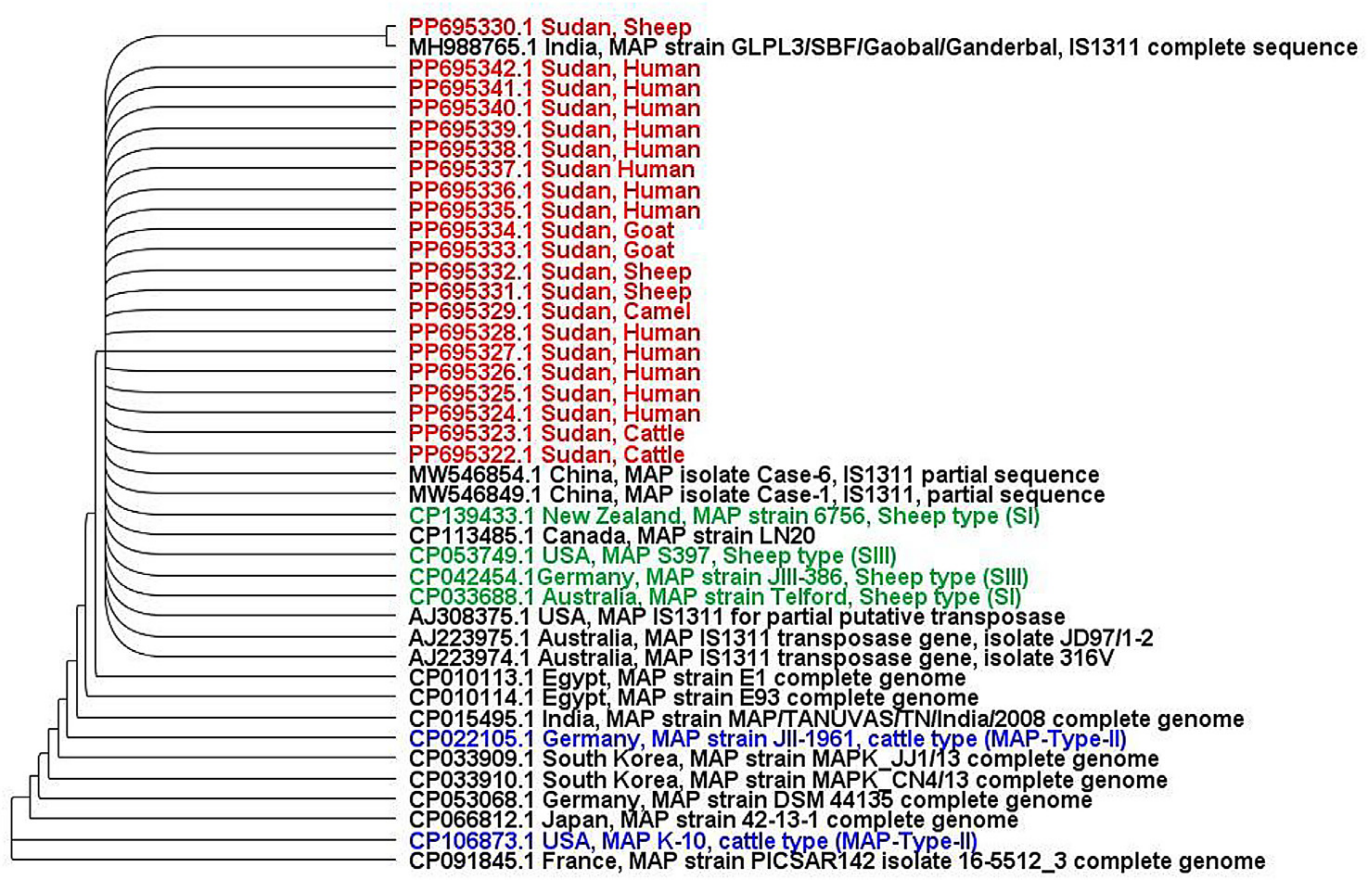
Maximum Likelihood phylogenetic tree based on partial sequences of the *Mycobacterium avium* subspecies *paratuberculosis* (MAP) 1311 insertion element of the Sudanese human isolates with corresponding sequence of other strains from the Sudan and worldwide. The Geneious programme (Geneious version 10.0, Biomatters, available from http://www.geneious.com) was used to construct the tree with 500 bootstrap replicates.

For this purpose, the constructed phylogenetic tree includes sequences of MAP strains from GenBank, representing different host species and countries (Australia, the USA, Germany, New Zealand, India, etc.). These strains included both Sheep-type (S-type) and Cattle-type (C-type) genotypes. Phylogeny results revealed that all Sudanese human isolates exhibited close genetic relationships to Sudanese animal MAP isolates and to global S-type (Sheep-type) strains from Australia, the USA, Germany, and New Zealand. Conversely, while revealing a distant genetic relationship to the K-10 reference strain and other strains, which belong to the C-type strain (Figure 1).

Two supplementary tables were constructed to summarize the BLAST results of the sequences of the Sudanese human MAP isolates aligned with those of MAP strains in the GenBank. Table 1 presents strains that exhibited close phylogenetic relatedness to the Sudanese human isolates, while Table 2 includes strains that were more distantly related. Both tables contain the same data categories: strain name, identity percentage, country of origin, GenBank accession number, animal host, and genotype classification.

**Table 1:** Summary of the BLAST results of the nucleotide sequences of the Sudanese human *Mycobacterium avium* subspecies *paratuberculosis* (MAP) isolates aligned to those of MAP strains from other countries showing close genetic relationships.

Strain	Identity %	Country	GenBank Accession Number	Animal Host	Genotype
MAP 6756	99.55	New Zealand	CP139433.1	Sheep	SI
MAP LN20	99.55	Canada	CP113485.1	Sus scrofa domesticus	NA
Telford	99.55	Australia	CP033688.1	Sheep	SI
MAP JIII-386	99.55	Germany	CP042454.1	Sheep	SIII
MAP S397	99.55	USA	CP053749.1	Sheep	SIII
Isolate Case-6	98.99	China	MW546854.1	Sheep	NA
Isolate JKSMAP-52	99.05	India	OR885898.1	Sheep	NA

**Table 2:** The BLAST results of the sequences of the Sudanese human *Mycobacterium avium* subspecies *paratuberculosis* (MAP) isolates revealed a distant genetic relationship with MAP strains documented in the GenBank database.

Strain	Identity %	Country	GenBank Accession number	Animal Host	Genotype
K-10	99.55	USA	CP106873.1	Cattle	C-type
JII-1961	99.55	Germany	CP022105.1	Cattle	C- type
E1	99.11	Egypt	CP010113.1	Cattle	NA

## 4 Experimental Design, Materials and Methods

### 4.1 Culture methods for MAP isolation

Collection of human samples was made after ethical approval by the Ethical Committee of Ibn Sina Specialized Hospital (ID:18,022,018) and written informed consent of the patients. A total of 45 stool samples and 62 tissue samples were subjected for culture. A decontamination step was done for all samples using sterile solution of 0.75 % hexadecyl pyridinium chloride (HPC) for 24 h at room temperature following standard decontamination protocols of MAP culture as described by Whittington et al. [18], before being inoculated on Middlebrook 7H11 (Merck, Darmstadt, Germany) agar slants supplemented with vancomycin, nalidixic and amphotericin B (VAN), oleic acid- albumin-dextrose-catalase (OADC) and mycobactin J (2 mg/L, Allied Monitor, USA). The inoculated slants were incubated at 37 °C, checked after 4 weeks and then every month for up to 20 months for visible growth.

### 4.2 Extraction of MAP DNA from culture

Colonies were collected from the slant surfaces of MAP positive cultures using a wire loop. These colonies were then emulsified in Tris EDTA buffer (pH 8.0) within 2 ml Eppendorf tubes. The samples were heated at 95 °C for 15 min, followed by centrifugation at 3000 rpm for 10 min. The supernatant was carefully decanted and stored at – 20 °C until being used as DNA template in PCR [19].

### 4.3 Nested- polymerase chain reaction (nPCR) of the IS1311 element

This assay was described by Whittington et al. [20]. Briefly, the Maxime premix (Intron Seol, Korea) was adjusted to 20 µl a total reaction volume with 13 µl molecular biology grade H_2_O, 1 µl (10 pMol/ µl) of each primer and 5 µl of DNA template for the first PCR round. For the second PCR round, 17 µl of H_2_O and 1 µl of the primers as well as the PCR product were used. The primer set M56 F: 5`- GCG TGA GGC TCT GTG AA-3`; M62: 5`- GCC TAT TTG CAC GGC ACC TC-3` was used for the first PCR, and the set M57 5`- GAT TGG TCG GCT GAA TCG GA-3`; M63 5`- GAT CCC TTG GGC ACC TGG GC-3`) was for the second PCR round. The following conditions were used for both PCR rounds: one cycle of denaturation at 94 °C for 2 min followed by 37 cycles of denaturation at 94 °C for 30 s, annealing at 62 °C for 15 s and extension at 72 °C for 1 min. The PCR amplification products were evaluated by electrophoresis at 100 V in 1 % agarose gels stained with SYBR safe stain (Invitrogen, Carlsbad, CA, USA), using100 bp DNA ladder as size marker.

### 4.4 Sequencing of IS1311 gene and phylogenetic analysis

The amplified products of the nested PCR were sent to a commercial company (Macrogen-Europe, Amsterdam, the Netherlands) for sequencing using the nested primers. Sequences of high-quality were edited using BioEdit programme [21]. Edited sequences were aligned to available MAP strains in NCBI GenBank using the Basic Local Alignment Search Tool (BLAST).

The phylogenetic tree was constructed using the sequences of human isolates with corresponding sequences of MAP strains from different countries obtained from the GeneBank. The Geneious programme (Geneious version 10.0, Biomatters, available from http://www.geneious.com) was used to construct the tree with 500 bootstrap replicates.

## Limitations

Not applicable.

## Ethics Statement

The authors declare that the work included human subjects from which the relevant informed consent was obtained. Collection of human samples was made after ethical approval by the Ethical Committee of Ibn Sina Specialized Hospital, the Sudan (ID:18,022,018).

## Credit Author Statement

Conceptualization: Kamal H. Eltom, Sanaa M. Idris, Wisal A. Elmagzoub Julius B. Okuni, Lonzy Ojok, Ahmed Abd El Wahed, ElSagad Eltayeb.

Data curation: Sanaa M. Idris, Wisal A. Elmagzoub, Maha Isameldin, Ahmed Abd El Wahed, Kamal H. Eltom.

Funding acquisition: Julius B. Okuni, Ahmed Abd El Wahed, Ahmad Amanzada, Kamal H. Eltom, ElSagad Eltayeb.

Investigation: Sanaa M. Idris, Wisal A. Elmagzoub, Maha Isameldin, Nassir Arabi, Abdelmonem Abdo, Mustafa Ibrahim, Kamal H. Eltom, ElSagad Eltayeb.

Methodology: Sanaa M. Idris, Wisal A. Elmagzoub, Julius B. Okuni, Lonzy Ojok, Ahmed A. Gameel, Ahmed Abd El Wahed, Kamal H. Eltom, ElSagad Eltayeb.

Project administration: Ahmed Abd El Wahed, Ahmad Amanzada, Kamal H. Eltom, ElSagad Eltayeb.

Resources: Maha Isameldin, Nassir Arabi, Abdelmonem Abdo, Mustafa Ibrahim, Julius B. Okuni, Ahmed Abd El Wahed, Ahmad Amanzada, ElSagad Eltayeb.

Supervision: Kamal H. Eltom, Ahmed Abd El Wahed, ElSagad Eltayeb, Uwe Truyen.

Validation: Sanaa M. Idris, Wisal A. Elmagzoub.

## Data Availability

Mendeley DataSequences of Mycobacterium avium subsp. paratuberculosis isolated from patients in the Sudan (Original data).
